# Transcriptomic and Metabolomic Insights into the Hepatic Response to Dietary Carvacrol in Pengze Crucian Carp (*Carassius auratus* var. Pengze)

**DOI:** 10.3390/genes16121491

**Published:** 2025-12-13

**Authors:** Wenshu Liu, Yuzhu Wang, Xiaoze Guo, Jingjing Lu, Lingya Li, Siming Li, Yanqiang Tang, Haihong Xiao

**Affiliations:** 1 Institute of Animal Husbandry and Veterinary Medicine, Jiangxi Academy of Agricultural Sciences, Nanlian Road, Nanchang 330200, China; wangyuzhu@jxaas.cn (Y.W.); guoxz@jxaas.cn (X.G.); lujingjing@jxaas.cn (J.L.); lilingya@jxaas.cn (L.L.); tangyqq@jxaas.cn (Y.T.);; 2 Jiangxi Province Key Laboratory of Animal Green and Healthy Breeding, Nanchang 330200, China

**Keywords:** carvacrol, pengze crucian carp, liver, transcriptomic, metabolomic

## Abstract

Background/Objectives: Carvacrol, a major active component of oregano oil and common feed additive, has been widely studied for its effects on fish growth, immunity, and intestinal health. But its transcriptional/metabolic impacts on fish liver remain unclear. This study investigated these effects in Pengze crucian carp (*Carassius auratus* var. Pengze). Methods: Fish were fed a basal diet (control) or basal diet supplemented with 10% microencapsulated carvacrol (600 mg/kg) for 56 days; liver samples were analyzed via transcriptomics and metabolomics. Results: Transcriptomic analysis revealed 482 differentially expressed genes (DEGs) in the liver of Pengze crucian carp following carvacrol supplementation, with 158 upregulated and 324 downregulated genes. Functional annotation highlighted enrichment in translation, signal transduction, amino acid metabolism, and posttranslational modification pathways. GO analysis further identified key processes, including carboxylic acid transport, tRNA aminoacylation, and mitochondrial nucleoid function, while KEGG pathways were implicated in amino acid biosynthesis, lipid metabolism (e.g., alpha-linolenic acid), and insulin signaling. Metabolomic profiling identified 679 significantly altered metabolites, including 113 upregulated and 566 downregulated ones. Among these, upregulated compounds like L-asparaginyl-L-lysine (Log_2_FC = 4.36) and 2′-Deoxyadenosine-5′-diphosphate (Log_2_FC = 4.31) are linked to nucleotide metabolism, and downregulated peptides (e.g., Ala-Phe-Tyr-Arg) suggesting modulated protein turnover. Joint omics analysis revealed convergent pathways in glycerophospholipid metabolism, aminoacyl-tRNA biosynthesis, and autophagy. Notably, the chaperone gene *dnaja3b* was correlated strongly with neuroactive metabolites (e.g., normetanephrine), potentially implicating carvacrol in stress response regulation. Conclusions: Our findings demonstrate that carvacrol modulates liver gene expression and metabolic profiles, primarily influencing amino acid and lipid metabolism pathways, autophagy, and stress responses. The observed correlations between *dnaja3b* and specific metabolites offer mechanistic insights into the action of carvacrol in fish liver.

## 1. Introduction

Since the 1950s, antibiotics have been widely used as feed additives in livestock farming due to their selective antibacterial properties and minimal adverse effects on animal somatic cells [1]. Beyond disease prevention, they enhance feed conversion efficiency and modulate immune responses by regulating gut microbiota and suppressing gastrointestinal infections [2]. Yet their extensive use has raised serious concerns in the scientific community [3,4]. A major issue is the incomplete absorption of veterinary antibiotics, leading to their excretion into aquatic and terrestrial environments [5,6], which accelerates the emergence and spread of antibiotic-resistant bacteria (ARB) [7]. ARB have since evolved into a global public health crisis.

In response, many countries and organizations have imposed bans on antibiotic use in animal husbandry [8,9,10]. Yet this reduced antibiotic use has led to unintended consequences, including decreased animal productivity and increased mortality rates [11]. Consequently, finding effective alternatives to antibiotics that sustain growth performance has become a critical research focus in the livestock industry. Among potential solutions, immunostimulants with strong antibacterial properties have emerged as a promising strategy [12,13].

Immunostimulants are designed with the goal of improving fish health and performance in aquaculture without causing harm [14]. As such, they have become a key research focus in feed hygiene, animal health, and additive development. Carvacrol (5-isopropyl-2-methylphenol), the primary bioactive compound in oregano oil, is a well-documented immunostimulant. Studies demonstrate its multifaceted benefits in aquaculture, including antibacterial effects [15,16], growth promotion [17], and immune enhancement [18,19].

While existing research highlights carvacrol’s regulatory effects on fish intestinal health [20,21], metabolism [22], and immunity [23], most studies focus on gut microbiota, with limited attention given to the liver—a vital metabolic and immune organ in fish that not only exerts core immune functions by producing acute-phase proteins and detoxifying inflammatory by-products [24], but also maintains close crosstalk with metabolic regulation by coordinating energy allocation during immune activation [25]. Although carvacrol has been shown to exert pro-inflammatory and apoptotic effects on liver tissue of LPS induced inflammation in fish [26], its transcriptomic and metabolomic impacts on fish liver remain unexplored. Pengze crucian carp (*Carassius auratus* var. Pengze), a premium aquaculture species developed in the 1980s by Jiangxi and Jiujiang Fisheries Research Institutes, is prized for its tender meat, high nutritional value, and strong production performance. Yet intensive farming practices in China increase its susceptibility to disease outbreaks such as bleeding disease caused by *Aeromonas hydrophila*, necessitating effective health management strategies. In our previous research, we have previously investigated the effects of different dietary inclusion levels (0, 200, 400, 600 mg/kg) of microencapsulated carvacrol on growth performance, intestinal barrier function (including microbial, physical, and immune barriers), and disease resistance in Pengze crucian carp, but neglected its impact on the liver [20]. Therefore, this study aimed to investigate the dynamic changes in the liver transcriptome and metabolome on metabolism and immune response of Pengze crucian carp fed carvacrol-supplemented diets, providing insights for its application as an antibiotic alternative in aquaculture.

## 2. Materials and Methods

### 2.1. Fish Husbandry

The juvenile Pengze crucian carp utilized in this experiment were sourced from a Pengze crucian carp breeding base in Jiujiang City, Jiangxi Province. Prior to the feeding trial, the fish underwent a two-week acclimation period at the aquaculture laboratory of the Jiangxi Academy of Agricultural Sciences, and both the acclimation and feeding trials were conducted in aquariums (60.0 × 60.0 × 80.0 cm) equipped with a recirculating filtration system. The system comprises a 50 mm filter sponge filter layer (filtration precision: 100 μm), a 20 mm activated carbon layer (particle size: 0.5 mm), a temperature control module, an aeration auxiliary module, and a water circulation rate of 2.5 times per hour.

### 2.2. Experimental Design and Sampling

According to our previous experimental method and results, all the feed ingredients were crushed into powder that could pass through a 200-mesh sieve, and then, 0 or 600 mg/kg of microencapsulated carvacrol (10%) (Jiangxi Tianjia Biological Engineering Co., Ltd., Nanchang, China) was added to the feed and thoroughly blended (the CK group (control check group) had no addition, while the CA group (carvacrol group) had 600 mg/kg of microencapsulated carvacrol). The 600 mg/kg dosage was selected because it demonstrated superior efficacy in enhancing disease resistance and improving intestinal health [20]. Table 1 presents the formulation and chemical compositions of the basal and experimental diets. The feed blend was then processed into pellets using a manual pasta machine, followed by air-drying at ambient temperatures ranging from 24 to 31 °C. Subsequently, the large pellets were crushed into small particles, and appropriately sized particles were collected through sieving (40-mesh). Finally, the prepared experimental feed was stored at −20 °C for future use.

A total of six aquariums were used in this experiment, with three aquariums each assigned to the CK and CA groups. Ten fish of uniform size (5.63 ± 0.35 g) were randomly distributed into each aquarium. During the 8-week feeding trial period, the fish were manually fed to apparent satiation twice daily (at 09:00 and 15:00). The initial feeding rate was set at 3% of the fish’s body weight and adjusted accordingly based on their feeding behavior. Throughout the experiment, one-third of the water in each aquarium was replaced daily with isothermal aerated water, and continuous aeration was provided using an air pump. Daily water quality monitoring was performed to ensure that the following conditions were maintained: pH (6.5–7.6) and temperature (27.8 ± 0.9 °C) measured by a portable pH meter (Model PHBJ-260, Shanghai INESA Scientific Instruments Co., Ltd., Shanghai, China), dissolved oxygen (>6.20 mg/L) determined with a portable dissolved oxygen meter (Model JPBJ-610L, Shanghai INESA Scientific Instruments Co., Ltd., Shanghai, China), and nitrate (<0.08 mg/L) measured using a water quality analyzer (Model DGB-480, Shanghai INESA Scientific Instruments Co., Ltd., Shanghai, China).

Upon completion of the 8-week feeding trial, all the fish groups in the experimental sets underwent a 24-h fasting period. Following this, 10 fish were randomly selected from each group for sampling. The fish were rapidly stunned by means of a rapid strike to the head, and their livers were dissected and separated. Each group had a total of 5 test samples, with each test sample being pooled from two liver samples of the same group for subsequent detection. These pooled liver samples were rapidly frozen with liquid nitrogen and then stored in a −80 °C freezer for subsequent testing. The growth performance data of the fish were presented in our previous report [20].

### 2.3. Cytokine Levels and Antioxidant Capacity

Liver cytokines levels, including TNF-α, IL-1β, and IL-10, were measured using commercially available ELISA kits according to the manufacturer’s instructions (Meimian-BIO, Suzhou, China). The antioxidant capacity of liver tissue was also assessed. Activities of catalase (CAT), glutathione peroxidase (GSH-Px), total antioxidant capacity (T-AOC), and superoxide dismutase (SOD) were determined using corresponding assay kits (Aidisheng-Bio, Yancheng, China). Reactive oxygen species (ROS) intensity were quantified with a fluorescence-based kit (Geruisi-Bio, Suzhou, China). Statistical analysis was performed with an independent samples *t*-test, and results were visualized using GraphPad Prism 9.5 (GraphPad Software, La Jolla, CA, USA).

### 2.4. Transcriptomic Analysis

#### 2.4.1. RNA Extraction

We isolated total RNA from liver specimens utilizing the TRIzol reagent (Thermo Fisher Scientific, Waltham, MA, USA). Approximately 50–100 mg of tissue was pulverized in 1 mL of TRIzol. Subsequent steps involved chloroform-mediated phase separation and isopropanol-induced RNA precipitation. The resulting RNA pellet underwent washing with 75% ethanol, was air-dried, and finally resuspended in RNase-free water. RNA integrity and potential contamination were evaluated via 1% agarose gel electrophoresis (Thermo Fisher Scientific, Waltham, MA, USA). We assessed RNA purity and concentration with a NanoPhotometer spectrophotometer (Implen GmbH, Munich, Germany) and the Qubit^®^ RNA Assay Kit (Thermo Fisher Scientific, Waltham, MA, USA) on a Qubit^®^ 2.0 Fluorometer (Thermo Fisher Scientific, Waltham, MA, USA), respectively. Furthermore, RNA integrity was verified employing the RNA Nano 6000 Assay Kit (Agilent Technologies, Santa Clara, CA, USA) on an Agilent Bioanalyzer 2100 system (Agilent Technologies, Santa Clara, CA, USA).

#### 2.4.2. Library Preparation for Transcriptome Sequencing

Sequencing libraries were prepared from 1 µg of total RNA per sample according to the protocols provided with the NEBNext^®^ Ultra™ RNA Library Prep Kit (New England Biolabs, Inc., Ipswich, MA, USA). Each library was labeled with a unique index code for sample multiplexing. The final library quality was verified using the Agilent Bioanalyzer 2100 system.

#### 2.4.3. Clustering and Sequencing

Indexed libraries underwent cluster generation on the Illumina cBot Cluster Generation System (Illumina, San Diego, CA, USA) using the TruSeq PE Cluster Kit (Illumina, San Diego, CA, USA). The pooled libraries were first denatured and then diluted to 8 pM with ice-cold hybridization buffer, followed by the addition of 1% (*v*/*v*) PhiX Control library. This prepared mixture was introduced into a flow cell, where bridge amplification was carried out on the cBot instrument under the specified thermal conditions: initial denaturation at 98 °C for 30 s; 10 cycles each of denaturation at 98 °C for 10 s, annealing at 60 °C for 30 s, and extension at 72 °C for 30 s; and a final extension at 72 °C for 5 min. After clustering, the flow cell was moved to an Illumina sequencer for paired—end sequencing based on cyclic reversible termination chemistry. Critical run parameters such as Q30 scores (consistently exceeding 80%) and cluster density were tracked throughout the process. Real-time base calling from raw image data was conducted with Illumina’s RTA software v2.7.1. The resulting data were demultiplexed and transformed into FASTQ files via bcl2fastq, generating 150 bp paired-end reads.

#### 2.4.4. Data Processing

Clean reads derived from raw sequencing data via adapter removal and quality filtering with fastp were mapped to the *C. auratus* reference genome (GCF_003368295.1_ASM336829v1_genomic.fna, GCF_003368295.1_ASM336829v1_genomic.gff) with HISAT2 to determine their genomic coordinates. Differential gene expression across experimental conditions was assessed using the DESeq2 package. To control the false discovery rate, *p*-values from the analysis were corrected via the Benjamini–Hochberg method. Transcripts satisfying the criteria of |log_2_(fold change)| ≥ 1 and an adjusted *p*-value (FDR) ≤ 0.05 were identified as significantly differentially expressed.

#### 2.4.5. RT-qPCR Validations of RNA-Seq Data

To verify the RNA—seq results, RT—qPCR was performed to examine the expression of the following genes: *dnaja3b*, *abcd2*, *tars1*, *mthfd2*, *slco5a1*, and *crybg2*. Total RNA was extracted from three fish per treatment using the TRIzon^®^ Reagent Up Plus RNA Kit (CWBIO, Shanghai, China) following the manufacturer’s protocol. Reverse transcription was carried out with the NovoScript^®^ Plus All-in-one 1st Strand cDNA Synthesis SuperMix (gDNA Purge) (Novoprotein, Shanghai, China) as instructed.

Specific qPCR primers (Table 2) were carefully designed based on sequences retrieved from GenBank using Primer 5 software (Premier Biosoft, Palo Alto, CA, USA). Quantitative PCR was performed in a CFX Connect^TM^ Real-Time PCR instrument (Bio—Rad Laboratories, Shanghai, China) using Novostart^®^ Universal Fast SYBR qPCR SuperMix. Each 20 μL reaction contained 10 μL ChamQ Universal SYBR qPCR Master Mix (2×), 1 μL cDNA, 0.4 μL each of forward and reverse primers, and 8.2 μL ddH_2_O. The thermal cycling conditions were: 95 °C for 10 min, followed by 40 cycles of 95 °C for 10 s, 60 °C for 30 s, and 72 °C for 30 s. All reactions were run in triplicate. β-actin was used as the internal reference gene, and relative gene expression was calculated using the 2^−ΔΔCT^ method. Statistical analysis was performed with a paired *t*-test, and results were visualized using GraphPad Prism 9.5 (GraphPad Software, La Jolla, CA, USA).

### 2.5. Metabolome Analysis

#### 2.5.1. Sample Preparation and Extraction

Frozen samples (−80 °C) were thawed on ice and subsequently homogenized by grinding. A 20 mg aliquot of the homogenized tissue was combined with 400 μL of a pre-cooled extraction solution (methanol/water, 7:3, *v*/*v*) containing internal standards. The mixture was vigorously shaken, incubated on ice, and then centrifuged at 4 °C. The supernatant was collected, subjected to a freezing step at −20 °C to precipitate residual proteins, and centrifuged again. A 200 μL aliquot of the final supernatant was transferred for LC-MS analysis.

#### 2.5.2. HPLC Conditions

Separation was performed using gradient elution on a Waters ACQUITY Premier HSS T3 Column (Waters Corporation, Milford, MA, USA). The aqueous mobile phase (A) contained 0.1% formic acid, while the organic phase (B) consisted of acetonitrile with 0.1% formic acid. The following gradient profile was applied: an increase from 5% to 20% B within 2 min, followed by a rise to 60% B over the next 3 min, then a sharp increase to 99% B in 1 min, which was held for 1.5 min before quickly returning to the initial 5% B for column re-equilibration. Throughout the analysis, the column was kept at 40 °C, with a constant flow rate of 0.4 mL/min and an injection volume of 4 μL.

#### 2.5.3. MS Conditions (AB)

Data acquisition was conducted in information-dependent acquisition (IDA) mode utilizing Analyst TF 1.7.1 Software for instrument control. The electrospray ionization source was configured with both GAS1 and GAS2 at 50 psi, a curtain gas (CUR) pressure of 25 psi, and a source temperature of 550 °C. The declustering potential was set to ±60 V, and the ion spray voltage was +5000 V in positive ion mode or −4000 V in negative ion mode. Full-scan TOF MS data were collected across 50–1000 Da with a 200 ms accumulation time, with dynamic background subtraction active. For MS/MS acquisition, product ion spectra were recorded from 25–1000 Da using a 40 ms accumulation time. A collision energy of ±30 V (with a 15 V spread) was applied according to the polarity. Further IDA criteria included unit resolution, a charge state of +/−1, an intensity threshold exceeding 100 cps, exclusion of isotopes within 4 Da, a mass tolerance of 50 ppm, and a maximum of 18 candidate ions per cycle.

#### 2.5.4. Analysis of LC-MS Data

The acquired raw data files were first converted to mzML format. Subsequent processing, comprising peak detection, alignment across samples, and retention time adjustment, was executed with XCMS. Peak areas were normalized using the “SVR” algorithm. To ensure robustness, metabolic features absent in more than half of the samples within any experimental group were filtered out. Putative metabolite identification was achieved by matching against combined in-house and public repositories. Differential metabolites were defined by a Variable Importance in Projection (VIP) score greater than 1.0 (derived from OPLS-DA models generated in MetaboAnalystR) and a univariate *t*-test *p*-value below 0.05. Metabolites were annotated via the KEGG Compound database, and their potential biological roles were explored by mapping to the KEGG pathway database. Pathway enrichment significance was evaluated using a hypergeometric test.

## 3. Results

### 3.1. Changes of Cytokine Levels and Antioxidant Capacity

**Figure 1:**
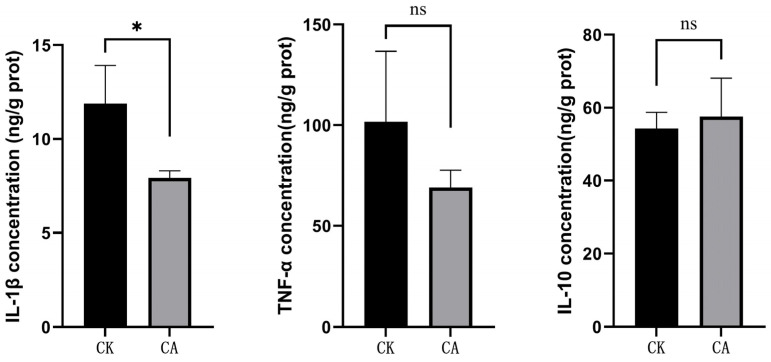
Cytokines level in liver of different experimental groups. *Note*: * *means p* < 0.05; ns *means p* > 0.05.

The results of liver cytokines levels, including IL-1β, TNF-α, and IL-10, are shown in Figure 1. The concentration of IL-1β was significantly lower in the CA group compared with the control group (*p* < 0.05). In contrast, no significant differences were observed in TNF-α or IL-10 levels between these two groups (*p* > 0.05).

**Figure 2:**
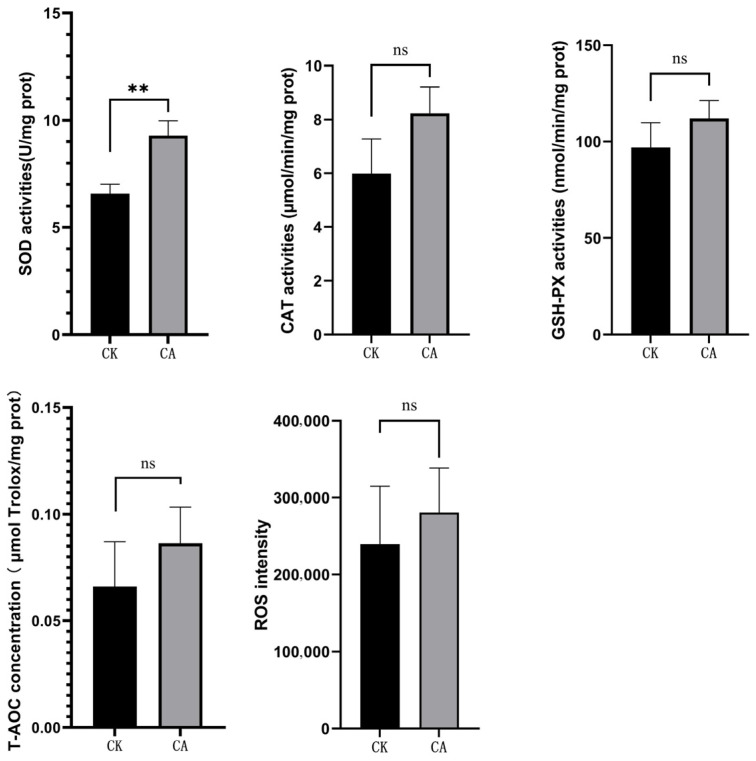
Liver antioxidant ability between different experimental groups. *Note*: ** *means p* < 0.01; ns *means p* > 0.05.

Regarding liver antioxidant capacity, the comparisons among experimental groups are presented in Figure 2. The activity of superoxide dismutase (SOD) was significantly higher in the CA group than that of the control group (*p* < 0.01). Although the activities of catalase (CAT), glutathione peroxidase (GSHPx), and total antioxidant capacity (T-AOC), as well as reactive oxygen species (ROS) intensity, showed an increasing tendency in the CA group, these changes did not reach statistical significance (*p* > 0.05).

### 3.2. Transcriptome Changes

#### 3.2.1. Transcriptome Sequence Assembly

Following data filtration, clean reads per sample varied from 42,845,296 to 56,624,310, derived from initial raw reads spanning 45,791,696 to 58,359,686. Total sequenced bases amounted to between 6.43 G and 8.49 G. The overall sequencing error rate remained low at either 0.02% or 0.03%. The proportion of bases achieving Qphred scores of at least 20 (Q20) and 30 (Q30) reached 97% and 93%, respectively, reflecting high base-calling accuracy. Furthermore, the GC content across samples ranged from 46.04% to 47.66% (Table 3). The complete raw RNA-seq dataset totals 70 GB. While these data are not publicly deposited due to privacy constraints, they can be requested directly from the corresponding author.

#### 3.2.2. Annotation and Function Analysis

Upon acquisition of clean data, alignment was performed against the reference genome of *C. auratus*. The resulting mapping statistics, including the total number of reads aligned to the genome, the count uniquely mapped, and those aligned to multiple positions, are summarized in Table 4. All samples demonstrated high mapping efficiency, with over 88.34% of reads aligned to the reference and more than 79.78% uniquely mapped.

#### 3.2.3. Changes in Gene Expression

**Figure 3:**
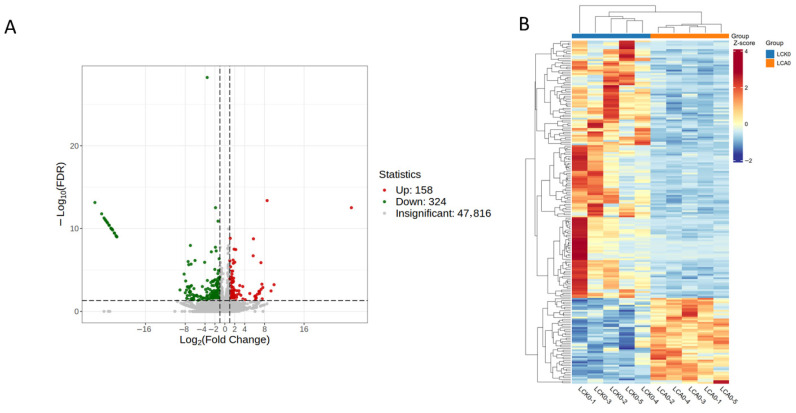
Volcano plot (**A**) and clustering heatmap (**B**) of differential genes between different experimental groups in liver of Pengze Crucian carp. Note: LCA and LCK represent liver samples from the carvacrol-treated and control group, respectively.

Transcriptomic analysis revealed substantial changes in gene expression within the liver. A total of 482 differentially expressed genes (DEGs) were identified between the control and treatment groups, consisting of 158 upregulated and 324 downregulated transcripts (Figure 3A). Cluster analysis based on a heatmap showed clear separation between the transcriptional profiles of the control and carvacrol (CA)-exposed groups (Figure 3B). This separation was statistically significant (PERMANOVA, *p* = 0.006), confirming that carvacrol treatment induces distinct alterations in the hepatic transcriptome.

**Figure 4:**
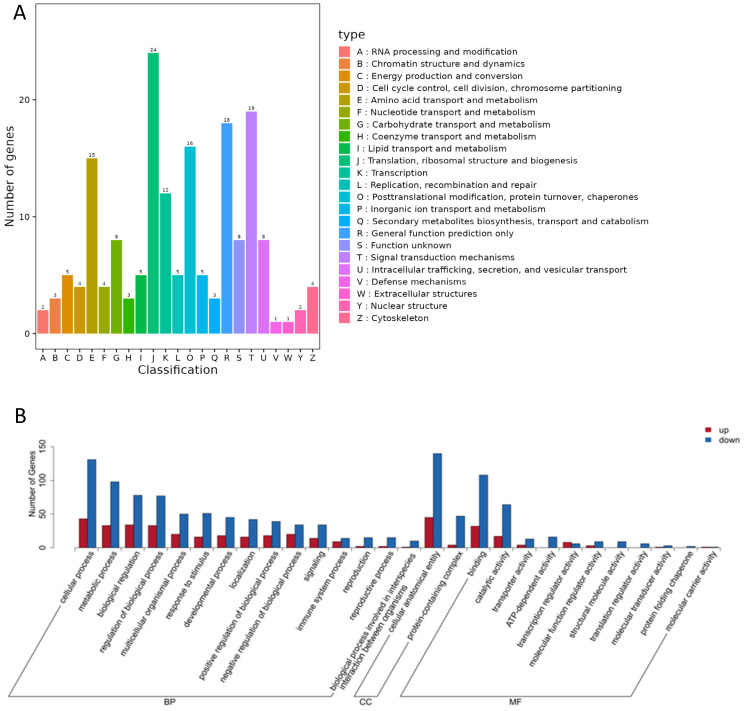
KOG annotation (**A**) and GO annotation (**B**) of differentially expressed genes in liver of Pengze Crucian carp.

Functional classification of the differentially expressed genes using the KOG (euKaryotic Orthologous Groups) database showed that, after removing entries labeled as “general function prediction only” or “function unknown,” the liver transcripts were assigned to 21 specific functional groups. The predominant categories include “Translation, ribosomal structure and biogenesis”; “Signal transduction mechanisms”; “Posttranslational modification, protein turnover, chaperones”; “Amino acid transport and metabolism”; and “Transcription” (Figure 4A, Table S2). Liver differentially expressed gene (DEG) GO enrichment analysis identified functionally distinct clusters across three major Gene Ontology (GO) categories—Biological Process (BP), Cellular Component (CC), and Molecular Function (MF)—with detailed annotations in Table S3 and the top 15 enriched terms visualized in Figure 4B.

**Figure 5:**
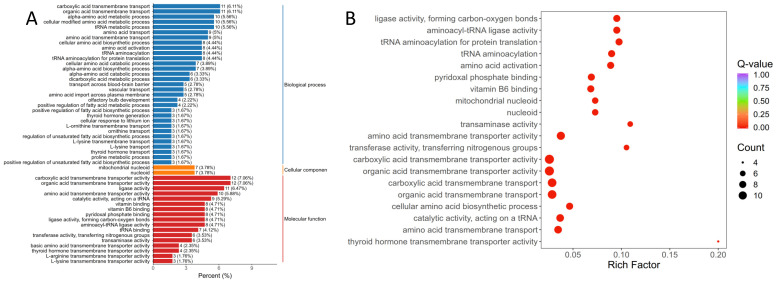
GO enrichment histogram (**A**) and sites (**B**) of differentially expressed genes in liver of Pengze Crucian carp of the different experimental groups.

The 50 terms with the smallest *q*-values used to generate the enrichment bar chart (Figure 5A); for KEGG enrichment, the 20 most significantly enriched pathways were plotted as a bubble map (Figure 5B), with full details (term description, gene ratio, counts of up/downregulated genes) in Table S4. Of the DEGs annotated to GO terms, 180 mapped to BP, 14 to CC, and 170 to MF, with enrichment prioritized for liver physiology and carvacrol-related functions: BP focused on amino acid metabolism/transmembrane transport (e.g., α-amino acid metabolic process, carboxylic acid transmembrane transport) and protein translation (e.g., tRNA metabolic process, tRNA aminoacylation for protein translation); CC was restricted to mitochondrial structural components (mitochondrial nucleoid, nucleoid), reflecting potential impacts on hepatic energy metabolism; MF centered on transport/catalytic activities tied to amino acid metabolism (e.g., amino acid transmembrane transporter activity, aminoacyl-tRNA ligase activity) and cofactor metabolism (vitamin B6, pyridoxal phosphate binding). The 20 most significantly enriched KEGG pathways reinforced these GO findings, primarily focusing on amino acid metabolism/transport (e.g., tRNA aminoacylation, amino acid activation) and secondarily covering hepatic cofactor metabolism, mitochondrial function, and thyroid hormone transmembrane transport—collectively illustrating carvacrol’s multi-faceted impacts on liver physiology.

**Figure 6:**
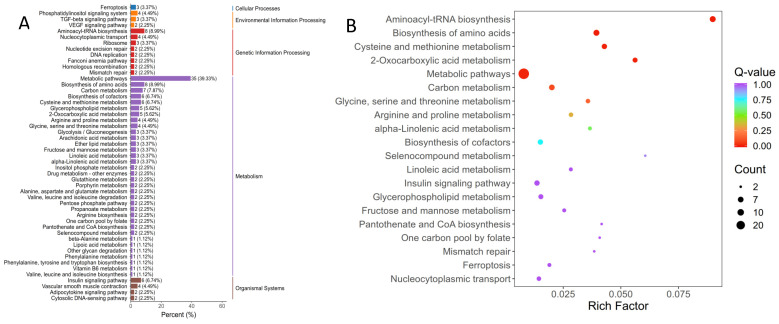
KEGG enrichment histogram (**A**) and sites (**B**) of differentially expressed genes in liver of Pengze Crucian carp of the different experimental groups.

The top 50 most significantly enriched pathways were used to generate a KEGG enrichment bar chart (Figure 6A), which displays both the number of DEGs assigned to each pathway and their proportion relative to all annotated DEGs. Dominant DEG annotations were distributed across pathways involved in Cellular Processes (3 genes), Environmental Information Processing (9 genes), Metabolism (89 genes), and Organismal Systems (14 genes). For more detailed visualization, a bubble chart was created based on the 20 most enriched pathways (Figure 6B), with comprehensive annotations including pathway description, gene ratio, and counts of upregulated and downregulated genes provided in Table S5. These analyses revealed that the DEGs were notably enriched in critical biological processes such as amino acid metabolism, energy and carbon metabolism, lipid metabolism, coenzyme and cofactor pathways, as well as signaling and regulatory networks.

#### 3.2.4. Expression Level of Validated Genes

**Figure 7:**
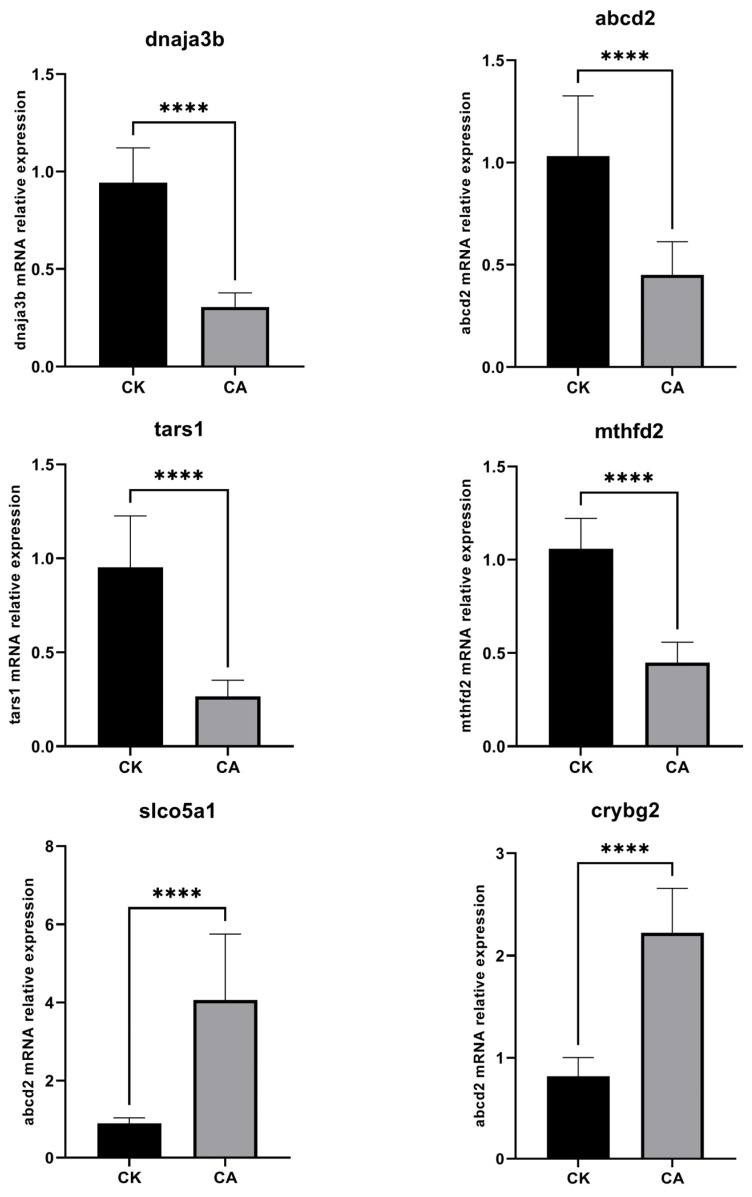
Expression levels of six genes. *Note*: **** *means p* < 0.001.

The expression levels of six genes (*dnaja3b*, *abcd2*, *tars1*, *mthfd2*, *slco5a1*, and *crybg2*) in the liver were validated by RT-qPCR (Figure 7). The results showed that the expression of *dnaja3b, abcd2, tars1*, and *mthfd2* was downregulated, whereas that of *slco5a1* and *crybg2* was upregulated, which is consistent with the RNA-seq findings.

### 3.3. Changes of Metabolic Profiles

**Figure 8:**
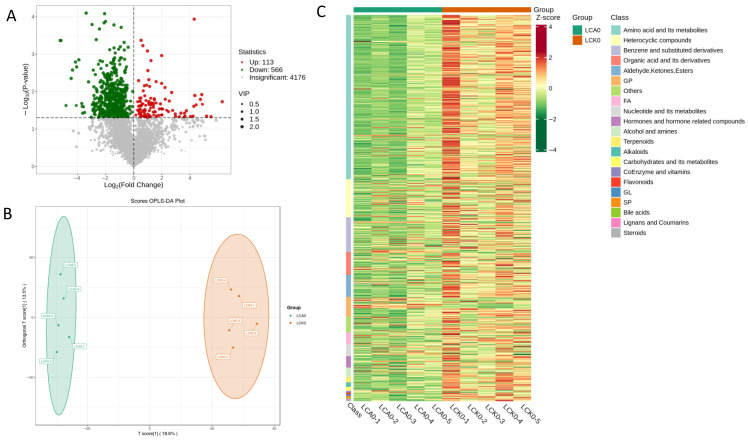
The metabolomic changes between different experimental groups in the liver of Pengze Crucian carp: (**A**) Volcano plot of differential metabolites; (**B**) OPLS-DA results of metabolite abundance; (**C**) clustering heatmap of differential metabolites in the different experimental groups.

In the liver samples, 4855 metabolites were detected, with 679 showing significant alterations relative to the control group. Among these differential metabolites in the carvacrol (CA)-treated Pengze crucian carp, 113 exhibited increased levels and 566 showed decreased levels (*p* < 0.05; Figure 8A). Comprehensive details regarding these metabolites are provided in Table S6. PLS-DA revealed a clear separation between the metabolic profiles of the carvacrol-treated and control groups, with samples clustering according to treatment, indicating that carvacrol significantly reshapes the liver metabolome in Pengze crucian carp (Figure 8B). Heatmap visualization revealed obvious segregation in metabolic profiles, with clear separation between the control and CA groups (Figure 8C), suggesting that carvacrol has caused statistically significant metabolic shifts in the metabolome of the liver tissues of Pengze crucian carp.

**Figure 9:**
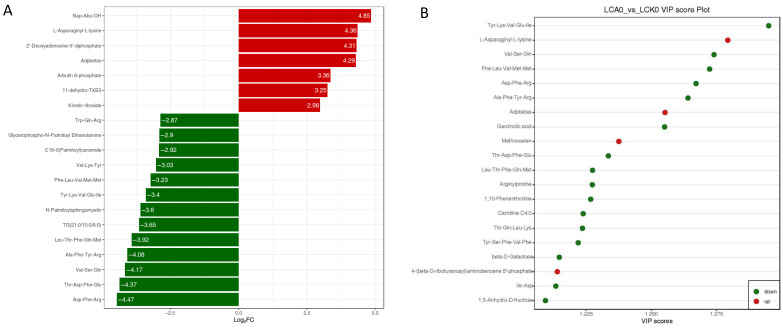
The metabolomic changes on Log_2_FC of differential metabolites (top 20) (**A**) and vipScore compounds (top 20) (**B**) between different experimental groups in the liver of Pengze Crucian carp.

Based on a univariate statistical analysis–fold change (FC) analysis between these two groups, a bar chart was generated to illustrate the top 20 metabolites exhibiting the most significant fold changes after eliminating the exogenous artificial compounds (Figure 9A). Several metabolites were elevated, including Nap-Abu-OH, L-Asparaginy-L-lysine, and 2′-Deoxyadenosine-5′-diphosphate, etc. In contrast, others such as Trp-Gln-Arg, Glycerophospho-N-Palmitoyl Ethanolamine, and C16-0 (Palmitoyl) ceramide showed reduced levels. Based on the orthogonal partial least squares-discriminant analysis (OPLS-DA), the variable importance in projection (VIP) values indicated that metabolites such as L-Asparaginyl-L-lysine, Adpbetas, and Methoxsalen were upregulated, whereas Tyr-Lys-Val-Glu-Ile, Val-Ser-Gln, and Phe-Leu-Val-Met-Met were downregulated (Figure 9B). By integrating the fold change (FC) with VIP results, it was further demonstrated that L-Asparaginyl-L-lysine and Adpbetas were significantly upregulated, while Tyr-Lys-Val-Glu-Ile, Asp-Phe-Arg, and Ala-Phe-Tyr-Arg exhibited notable downregulation.

**Figure 10:**
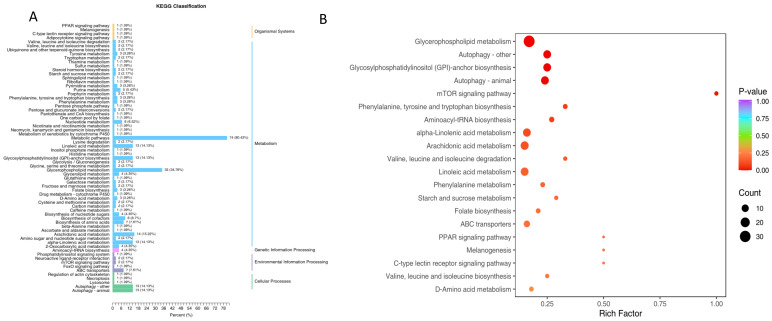
KEGG enrichment histogram (**A**) and sites (**B**) of differential metabolites in the liver of Pengze Crucian carp of the different experimental groups.

KEGG analysis of the significantly altered metabolites enabled their classification according to pathway categories defined in the KEGG database (Figure 10A). This functional classification highlighted a predominant enrichment within general metabolic pathways. Subsequent enrichment analysis, focused on the top 20 pathways ranked by *p*-value derived from the differential metabolite set, identified several key biological processes significantly perturbed by carvacrol (CA) treatment (Figure 10B). Compared to controls, the CA group showed marked enrichment (*p* < 0.05) in pathways related to lipid metabolism (including glycerophospholipid, alpha-linolenic acid, and arachidonic acid metabolism), amino acid metabolism (such as phenylalanine, tyrosine and tryptophan biosynthesis; valine, leucine and isoleucine degradation/biosynthesis; D-amino acid metabolism), autophagy and cellular stress responses (e.g., autophagy and the mTOR signaling pathway), as well as immune and signaling pathways like the C-type lectin receptor signaling pathway and GPI-anchor biosynthesis.

### 3.4. Conjoint Analysis

**Figure 11:**
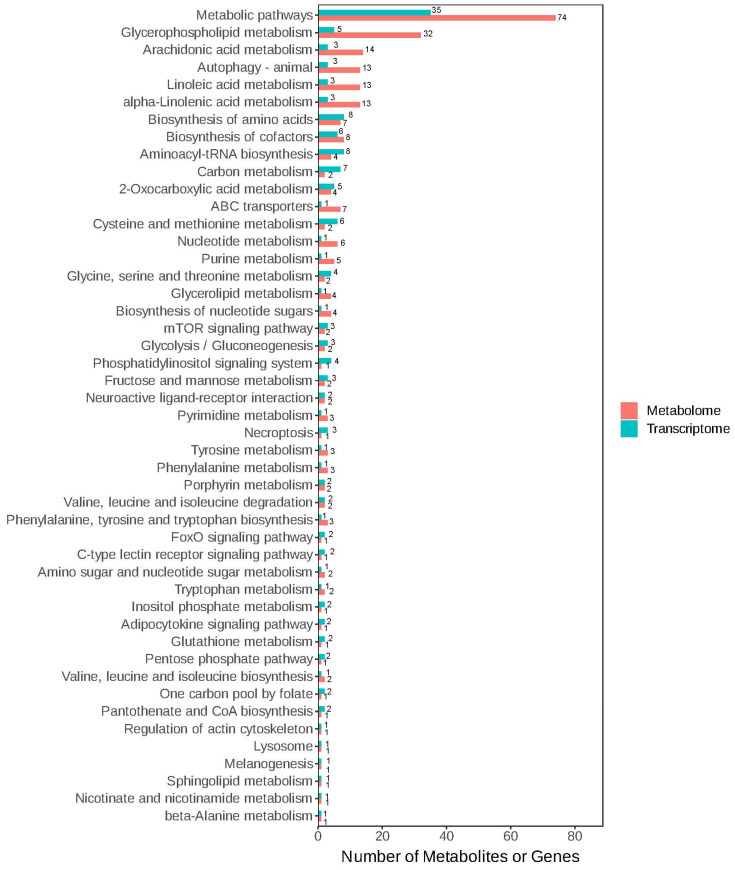
Metabolome and transcriptome KEGG co-enrichment bar graph in liver of Pengze Crucian carp.

Based on the differences in the metabolites of the liver, as well as the KEGG enrichment analysis results for the differential genes, the KEGG signaling pathways that were collectively enriched are shown in Figure 11. The KEGG pathways that were collectively enriched by both omics from the perspective of the number of differential genes and metabolites are ranked as metabolic pathways, glycerophospholipid metabolism, arachidonic acid metabolism, autophagy-animal, linoleic acid metabolism, and so on.

**Figure 12:**
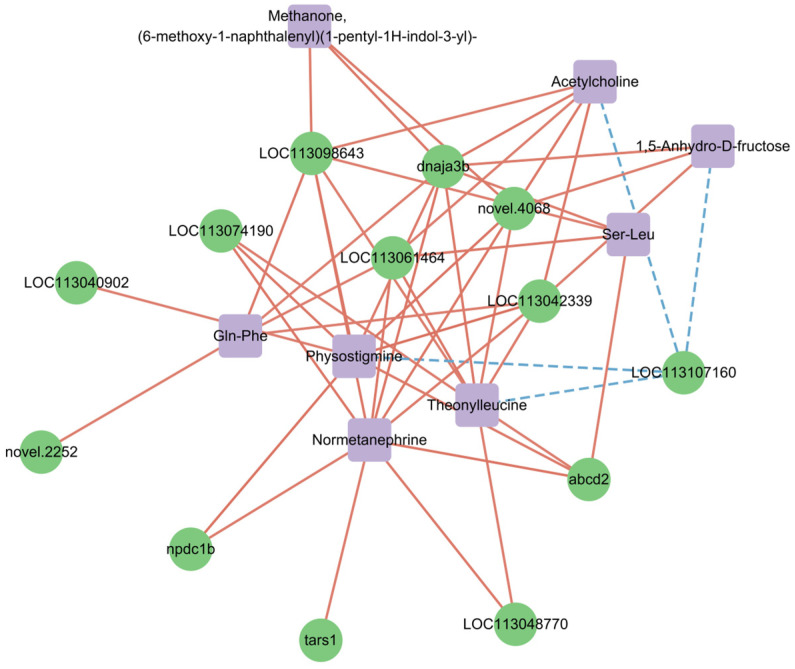
Network between genes and metabolites with significant correlation.

For the analysis of the correlation between differentially expressed genes and differentially metabolized substances, those with Pearson correlation coefficients greater than 0.80 and *p* < 0.05 were considered to have significant correlations. In the liver, the expression of genes such as *dnaja3b* was positively correlated with the levels of normetanephrine, Gln-Phe, methanone, (6-methoxy-1-naphthalenyl) (1-pentyl-1H-indol-3-yl), 1,5-Anhydro-D-fructose, Ser-Leu, physostigmine, acetylcholine, and theonylleucine. It is noteworthy that the gene *dnaja3b* is associated with all the levels of the above substances. The gene LOC113107160 was negatively correlated with the levels of 1,5-Anhydro-D-fructose, physostigmine, and theonylleucine (Figure 12, Table S7).

## 4. Discussion

Carvacrol, a prominent natural component of essential oils derived from aromatic plants, is known for its wide range of biological effects, including antioxidant, antimicrobial, antiviral, antidiabetic, cardioprotective, anti-obesity, hepatoprotective, reproductive-protective, anti-aging, and immunomodulatory activities [15,27]. As a crucial metabolic organ, the liver plays key roles in nutrient metabolism, detoxification, bile secretion, and immune regulation. It contains various immune cells—such as Kupffer cells, macrophages, melanomacrophages, and telocytes—that participate in phagocytosis, autophagy, and immune modulation [28]. Growing attention has been directed toward the impact of carvacrol and oregano oil extracts on the liver in livestock. Rodent studies have demonstrated that carvacrol exerts hepatoprotective effects by alleviating ischemia–reperfusion injury, as indicated by lowered ALT/AST levels, improved histological architecture, and the inhibition of apoptosis [29]. It also attenuates acrylamide-induced hepatotoxicity by inhibiting elevations in MDA, TOS, TNF-α, IL-1β, NF-κB, ALT, and AST, while maintaining tGSH and TAS concentrations [30]. In Akkaraman lambs, dietary supplementation with Orego-Stim (containing 5% essential oil of *Origanum vulgare* subsp. hirtum and 95% inert carrier) at doses of 200 and 400 mg/kg elevated hepatic catalase (CAT) activity and glutathione (GSH) levels [31]. Additionally, carvacrol was shown to inhibit hepatic stellate cell activation and proliferation in mice, thereby reducing liver fibrosis, potentially through modulation of the MAPK pathway [32]. Nevertheless, the effects of carvacrol or oregano oil on the livers of aquatic species remain unexplored. In the present study, administration of a diet supplemented with 600 mg/kg of microencapsulated carvacrol (10%) resulted in decreased IL-1β levels and significantly enhanced superoxide dismutase (SOD) activity in the liver of Pengze crucian carp. This demonstrates the compound’s efficacy in reducing liver inflammation and strengthening the antioxidant system. The present liver outcomes corroborate our earlier research [20] in which carvacrol was implicated in alleviating intestinal inflammation and improving gut health, collectively underscoring its systemic anti-inflammatory and antioxidant benefits.

RNA-seq technology provides a powerful tool for exploring natural growth promoters in animal nutrition. Although existing studies have investigated the transcriptional effects of oregano oil extracts on liver metabolism in terrestrial species such as chickens, pigs, and mice, the impacts of carvacrol or oregano oil on the hepatic transcriptomes of aquatic animals remain largely unexplored. In broilers, dietary supplementation with 2% oregano powder markedly altered hepatic gene expression, affecting steroid hormone regulation, and lipid and carbohydrate metabolism, and suppressing pathways related to hepatocellular carcinoma [33]. Similarly, broilers fed 0.2% aqueous oregano extract exhibited 129 differentially expressed genes (DEGs), with 104 downregulated and 25 upregulated. The downregulated genes were involved in fatty acid metabolism and insulin signaling, suggesting a potential role in reducing adiposity and managing metabolic disorders [34]. In weaned piglets, a phytogenic blend containing oregano oil counteracted transcriptomic changes caused by oxidized oil, particularly in genes related to fatty acid β-oxidation and peroxisomal pathways, indicating hepatoprotective and antioxidant benefits [35]. A study in rainbow trout revealed that dietary oregano essential oil upregulated immune-related genes (TLR3, MyD88, IRF7, TRAF2) and downregulated interleukin genes (IL-6, IL-21, IL-34) [36]. This collective evidence highlights a significant knowledge gap regarding the transcriptomic effects of carvacrol and oregano oil in aquatic species. In the present study, RNA-seq analysis identified 482 DEGs (158 upregulated, 324 downregulated) in the liver between the control and carvacrol-treated groups. GO enrichment analysis indicated substantial changes in pathways related to amino acid metabolism and transport, cofactor metabolism and binding (e.g., pyridoxal phosphate and vitamin B6 binding), mitochondrial nucleoid organization, carbon-oxygen ligase activity, and thyroid hormone transport. These transcriptomic alterations—particularly the enrichment of amino acid metabolism/transport pathways—may imply a potential role of carvacrol in regulating nutrient utilization (with a focus on amino acid use) and supporting protein synthesis, processes that have been associated with enhanced growth performance in aquatic species in previous studies [30,37,38]. Additionally, such alterations may also suggest a potential role in modulating cellular stress responses via autophagy [39]. KEGG enrichment analysis further revealed significant changes in metabolic and immune-related pathways, most notably in aminoacyl-tRNA biosynthesis, amino acid biosynthesis, cysteine and methionine metabolism, 2-oxocarboxylic acid metabolism, and carbon metabolism. These findings align with a previous trial in rainbow trout that also reported enrichment in amino acid metabolic pathways [36].

Metabolite variation serves as a key indicator of physiological status, environmental adaptation, and overall health in fish. The observed metabolic shifts likely arise from both direct actions of carvacrol and adaptive physiological reactions to its supplementation. Based on fold-change and variable importance in projection (VIP) criteria, compounds such as L-Asparaginyl-L-lysine and Adpbetas were upregulated. In contrast, several short peptides—including Tyr-Lys-Val-Glu-Ile, Asp-Phe-Arg, Ala-Phe-Tyr-Arg, Val-Ser-Gln, Leu-Thr-Phe-Gln-Met, and Thr-Asp-Phe-Glu—were downregulated. The downregulation of these short peptides in the liver with carvacrol may indicate an enhanced overall digestive function (i.e., increased ability to hydrolyze short peptides into free amino acids). These metabolite changes reflect a multifaceted metabolic reprogramming in the liver induced by dietary carvacrol. Functional analysis revealed that differential metabolites were significantly enriched in key biological processes such as lipid metabolism, amino acid metabolism, autophagy, cellular stress responses, and immune-related signaling pathways. This indicates that carvacrol exerts pleiotropic effects on the liver, including promoting lipid breakdown for energy, reducing hepatic fat deposition via PPAR signaling, potentially generating anti-inflammatory mediators [40,41], adapting to metabolic shifts through the mTOR pathway, and maintaining cellular homeostasis via autophagy. Supporting this, carvacrol was shown to have a protective effect against liver injury in mice, and the mechanism may be related to the inhibition of the mTOR inflammatory signaling pathway [42].

First identified in 1998 [43], DNAJA3 is a member of the evolutionarily conserved DNAJ/HSP40 protein family [44]. In humans, it interacts with various partners including viral proteins, heat shock proteins, and regulators of cell signaling and growth [45]. The fish ortholog *dnaja3b* retains similar functions—serving as a critical co-chaperone for Hsp70, maintaining proteostasis by facilitating the folding of nascent and stress-denatured proteins, preventing aggregation, and promoting the degradation of damaged proteins—while enhancing resistance to environmental stressors (notably thermal stress) to support embryonic development, tissue function (e.g., cardiac and muscular tissues), and overall cellular resilience [46]. In this study, multi-omics integration revealed that the expression of *dnaja3b* was positively correlated with the hepatic levels of normetanephrine; Gln-Phe; methanone derivatives (e.g., (6-methoxy-1-naphthalenyl) (1-pentyl-1H-indol-3-yl)); 1,5-Anhydro-D-fructose; Ser-Leu; physostigmine; acetylcholine; and theonylleucine. These correlations may point to a potential adaptive response in the liver, though this remains a hypothesis to be further validated—assuming these regulatory factors are produced within the liver. The association with both normetanephrine (a catecholamine metabolite) and acetylcholine may indicate concurrent activation of the sympathetic and parasympathetic systems, a pattern that has been linked to physiological stress in prior studies [47,48]. The upregulation of *dnaja3b* could potentially serve a protective role by ensuring the proper folding and functionality of stress response proteins, based on the observed correlations. Correlations with energy-related metabolites (e.g., 1,5-Anhydro-D-fructose) and dipeptides further indicate the restructuring of energy metabolism and protein turnover [49]. Additionally, links to methanone derivatives—which resemble endocannabinoid-like signaling molecules—hint at potential roles in inflammatory or lipid metabolic modulation [50], while correlations with physostigmine reinforce involvement in cholinergic anti-inflammatory pathways [51].

## 5. Conclusions

This study concludes that carvacrol alters the liver gene expression and metabolic profiles of Pengze crucian carp. Transcriptome analysis identified 482 differentially expressed genes, while metabolome analysis detected 679 differential metabolites. The integrated multi-omics analysis revealed that carvacrol primarily disrupts several key metabolic pathways–including amino acid, glycerophospholipid, and arachidonic acid metabolism–while also impairing mitochondrial function and autophagy processes. Central to these effects is the gene *dnaja3b*, which has been identified as a key target for further investigation.

## Figures and Tables

**Table 1:** Diet formulation and chemical composition of Pengze Crucian carp.

Ingredient	Percentage (%)	
	CK	CA
Fish meal	14.00	14.00
Soybean meal	26.00	26.00
Rapeseed meal	23.00	23.00
Corn grain	22.00	22.00
Soybean oil	2.00	2.00
Fish oil	1.00	1.00
Coated lysine	0.50	0.50
Coated methionine	0.25	0.25
Vitamin mixture ^a^	0.50	0.50
Mineral mixture ^b^	0.50	0.50
Choline	0.50	0.50
CaH_2_PO_4_	1.50	1.50
Vitamin C phosphate ester	0.10	0.10
Carboxymethyl cellulose sodium	2.00	2.00
Microcrystalline cellulose	6.15	5.55
Microencapsulated Carvacrol	0	0.60
Chemical composition		
Crude protein	33.14	33.15
Crude lipid	4.82	4.81
Crude ash	5.75	5.76
Moisture	10.2	10.1

Note: a, b The multi-dimensional and multi-mineral products used were purchased from Hefeng Livestock Co., Ltd, Shenyang, China.

**Table 2:** Sequences of oligonucleotide primers for qPCR.

Target Genes	Primers	Oligonucleotide (5′-3′)	References (NCBI Accession No.)
β-actin	*β-actin*-F	CTGGTATCGTGATGGACTCT	XM_026258408.1
	*β-actin*-R	AGCTCATAGCTCTTCTCCAG	
dnaja3b	*dnaja3b*-F	CAGTGTTTCGTCGTGATGGC	XM_026198116.1
	*dnaja3b*-R	GCCTGGAGGAATCGCAATGT	
abcd2	*abcd2*-F	AGATGCACATCAATGGCCCC	XM_026225352.1
	*abcd2*-R	CATCCCTTCCTCTACCTTGAAGT	
tars1	*tars1*-F	GCAAAGAGTGTCTGCTGAAATACC	XM_026211345.1
	*tars1*-R	GGAGGCCATAGCTTGGAAGAG	
mthfd2	*mthfd2*-F	CCCATGACCGTAGCCATGC	XM_026259937.1
	*mthfd2*-R	AGTGTGGAATGTGCAGGAGTTG	
slco5a1	*slco5a1*-F	GGATTCACCCACCAGGACAG	XM_026200927.1
	*slco5a1*-R	TCGGTTGGATTCAGTTCGCA	
crybg2	*crybg2*-F	GGGCTTTGCTGTGTCCCTAT	XM_026289848.1
	*crybg2*-R	TGACTCCTGGGCCTTCACTA	

**Table 3:** Results of transcriptomic sequence assembly of Pengze Crucian carp.

Sample	Raw Reads	Clean Reads	Clean Base (G)	Error Rate (%)	Q20 (%)	Q30 (%)	GC Content (%)
LCK0-1	45,791,696	42,845,296	6.43	0.02	98.51	95.51	47.05
LCK0-2	49,500,992	47,827,558	7.17	0.02	98.41	95.16	46.88
LCK0-3	46,497,432	45,024,468	6.75	0.02	98.50	95.39	47.00
LCK0-4	46,874,742	45,749,482	6.86	0.02	98.35	95.00	46.04
LCK0-5	46,562,158	45,104,346	6.77	0.03	97.93	93.93	46.68
LCA0-1	58,359,686	56,624,310	8.49	0.02	98.51	95.37	47.66
LCA0-2	46,806,092	45,612,338	6.84	0.02	98.47	95.31	46.37
LCA0-3	54,030,206	52,353,992	7.85	0.02	98.46	95.29	47.11
LCA0-4	49,461,430	47,833,286	7.17	0.02	98.45	95.26	46.85
LCA0-5	47,379,162	45,757,524	6.86	0.02	98.57	95.56	46.82

Note: LCA and LCK represent liver samples from the carvacrol-treated and control group, respectively. Q indicates the Qphred value.

**Table 4:** Comparison of efficiency statistics of Pengze Crucian carp.

Sample	Total Read Pairs	Total Mapped Reads	Uniq Mapped Reads	Multiple Mapped Reads
LCK0-1	42,845,296	38,651,367 (90.21%)	34,183,021 (79.78%)	4,468,346 (10.43%)
LCK0-2	47,827,558	43,101,583 (90.12%)	38,436,930 (80.37%)	4,664,653 (9.75%)
LCK0-3	45,024,468	40,645,941 (90.28%)	37,442,403 (83.16%)	3,203,538 (7.12%)
LCK0-4	45,749,482	40,413,797 (88.34%)	37,319,450 (81.57%)	3,094,347 (6.76%)
LCK0-5	45,104,346	40,729,223 (90.30%)	36,545,121 (81.02%)	4,184,102 (9.28%)
LCA0-1	56,624,310	51,227,129 (90.47%)	45,976,251 (81.20%)	5,250,878 (9.27%)
LCA0-2	45,612,338	40,862,162 (89.59%)	37,230,007 (81.62%)	3,632,155 (7.96%)
LCA0-3	52,353,992	47,370,453 (90.48%)	43,434,078 (82.96%)	3,936,375 (7.52%)
LCA0-4	47,833,286	43,238,485 (90.39%)	39,735,201 (83.07%)	3,503,284 (7.32%)
LCA0-5	45,757,524	41,165,238 (89.96%)	37,548,489 (82.06%)	3,616,749 (7.90%)

Note: LCA and LCK represent liver samples from the carvacrol-treated and control group, respectively.

## Data Availability

The data that support the findings of this study are available from the corresponding author upon reasonable request.

## Supplementary Materials

The following supporting information can be downloaded at: https://www.mdpi.com/article/10.3390/genes16121491/s1. Table S1: Internal standard of metabolome detection used in this study; Table S2: KOG classification of differentially expressed genes between the CA and CK groups; Table S3: GO classification of differentially expressed genes between the CA and CK groups; Table S4: GO enrichment of differentially expressed genes between the CA and CK groups; Table S5: KEGG enrichment of differentially expressed genes between the CA and CK groups; Table S6: Filtered different metabolites between the CA and CK groups; Table S7: Correlation analysis of differential genes and differential metabolites in liver.
